# Differences in equine spinal kinematics between straight line and circle in trot

**DOI:** 10.1038/s41598-021-92272-2

**Published:** 2021-06-18

**Authors:** A. Byström, A. M. Hardeman, F. M. Serra Bragança, L. Roepstorff, J. H. Swagemakers, P. R. van Weeren, A. Egenvall

**Affiliations:** 1 Department of Anatomy, Physiology and Biochemistry, Swedish University of Agricultural Sciences, 750 07 Uppsala, Sweden; 2 Tierklinik Luesche GmbH, Essenerstrasse 39a, 49456 Luesche, Germany; 3 Department of Clinical Sciences, Faculty of Veterinary Medicine, Utrecht University, Yalelaan 112-114, 3584 CM Utrecht, The Netherlands; 4 Department of Clinical Sciences, Faculty of Veterinary Medicine and Animal Science, Swedish University of Agricultural Sciences, 750 07 Uppsala, Sweden

**Keywords:** Anatomy, Bone quality and biomechanics

## Abstract

Work on curved tracks, e.g. on circles, is commonplace within all forms of horse training. Horse movements in circles are naturally asymmetric, including the load distribution between inner and outer limbs. Within equestrian dressage the horse is expected to bend the back laterally to follow the circle, but this has never been studied scientifically. In the current study 12 horses were measured (optical motion capture, 100 Hz) trotting on left and right circles and on the straight without rider (soft surface). Data from markers placed along the spine indicated increased lateral bending to the inside (e.g. left bending on the left circle) of the thoracolumbar back (difference left circle vs. straight − 3.75°; right circle + 3.61°) and the neck (left − 5.23°; right + 4.80° vs. straight). Lateral bending ROM increased on the circle (+ 0.87° and + 0.62°). Individual variation in straight-circle differences was evident, but each horse was generally consistent over multiple trials. Differences in back movements between circle and straight were generally small and may or may not be visible, but accompanying changes in muscle activity and limb movements may add to the visual impression.

## Introduction

When moving in circles in trot, the inside fore-and hind limbs take shorter strides (reduced pro- and retraction)^1^, are subjected to lower peak forces, and have longer stance duration than the outside limbs^2,3^. To move on a circle, the horse needs to accelerate its body laterally in the direction of the bend, or otherwise the horse would continue forwards tangential to the circle. To achieve this, the horse needs to create ground reaction force towards the centre of the circle. The magnitude of the force required depends on the velocity of the horse (v) and the radius (r) of the circle (transverse force needed = m*v^2^/r). This, however, presents a balance challenge. If the resultant force vector between the vertical and lateral horizontal ground reaction forces points to the inside of the horse’s centre of mass, it creates a moment that acts to tip the horse over to the outside^1^. To avoid this, the horse needs to shift its centre of mass towards the inside of the circle. The horse may do this by leaning into the circle. This strategy is well described^4–6^, with the degree of body lean (approximated as stride mean pelvis roll) being proportional to the horse’s speed (squared) and inversely proportional to the radius of the circle, in accordance with the theory of physics^4^. However, differences between predicted and observed body lean in individual horses suggest that the horse may use additional strategies to accomplish a lateral shift of its centre of mass.

Compared to a straight track, moving on a circle influences the horse’s vertical body movement symmetry, mimicking inside hind limb supporting lameness and inside forelimb lameness^7–9^: the croup is relatively less lowered during stance of the inside hind limb, and the withers are lowered relatively less during inside forelimb (outside hind limb) stance. The hip hike (tuber coxae upward movement) on the outer side, concurrent with inside hind limb stance, is reduced^7^; this may be (partly) related to changes in pelvic rotation, but that has not been investigated. Increased degree of body lean correlates with increases in all these vertical movement asymmetries suggestive of inside limb lameness^4^. With respect to head motion there is more individual variation^8,10^. The head is most often lowered relatively less during the inside forelimb—outside hind limb diagonal stance but head movement asymmetry indicative of outside forelimb lameness is not uncommon^11^. At group level, these asymmetries are reversed between left and right circles, but perfect mirroring is seldom the case in individual horses^11^.

Riding on circles, training the horse to bend laterally in the direction of the circle, is an important part of the horse’s basic schooling and aims at achieving both flexibility and straightness/symmetry of the horse, as it is perceived to stimulate the development of equal ability to bend to the left and to the right side^12^. It has been shown that horses working correctly in a dressage frame under a rider lean less inward than expected based on the speed and circle radius, suggesting these horses were able to use other means to maintain balance^13^. Conversely, horses awarded lower points for work quality tended to lean more than predicted^13^. Evaluating the horse on left and right circles is an important part of the lameness examination. Studies have shown additive effects between the asymmetry induced by the circular movement, and asymmetries related to lameness^8,9,14^ or to the rider performing rising trot^15–17^. Lame horses often show increased symmetry of body lean between left and right circles after successful diagnostic analgesia^14^. Back pain is known to alter back movement on a straight line^18,19^, but effects on circular motion have not been investigated. To further understand and correctly interpret the effects resulting from the interaction between moving in circles and all of the above-mentioned factors, it is important to understand more about how sound horses without a rider move in circles. For back movements, this includes normal stride ranges and movement characteristics, the degree of individual variation, and the normal range of asymmetries between left and right directions.

Several previous studies have described back movements in sound unridden horses moving straight on treadmill^20–22^, or comparing treadmill and over-ground^23^. For circle only range of motion has been studied, which increased compared to straight line for both lateral bending and flexion-extension^5,24^. There are, however, no reports on the extent to which horses show (inside) lateroflexion of the vertebral column when moving in circles, nor if altered flexion–extension of the back plays a role in adaptation to circular movement. As follows, relationships between body lean and back movements have not been investigated. The aim of the present study was to describe both group and individual patterns (multiple measurements of each individual) in movements of the equine back and neck on the circle compared to the straight. The study focused on flexion–extension and lateral bending of the thoracolumbar back, pelvic rotations, head lateral position relative to the body (head swivel/cervical lateral bending), and tracking of the hindquarters relative to the forehand. The hypothesis was that ROM and/or stride mean for these kinematic variables would differ significantly compared to straight line, in adaptation to circular movement. It was further hypothesized that changes in flexion–extension and lateral bending of the thoracolumbar back would correlate with changes in pelvic rotations, cervical lateral bending, tracking of the hindquarters and/or body lean.

## Materials and methods

### Horses

Twelve privately owned horses were recruited to the study. All horses were deemed sound by their owners/trainers, performing well during training, and not known to have neck or back dysfunction^25^. Age range was 5–15 years, mean 8.3 years, and body mass 450–652 kg (mean 551 kg). There were 11 European warmbloods and one Friesian, three geldings and nine mares. Competition level varied from not competing up to intermediate level in either show jumping or dressage. All experiments were performed in accordance with relevant guidelines and regulations. According to German law and regulations (Tierschutzgesetz §7), ethical approval is not required for non-invasive experiments where the studied animals are not subjected to any additional risks, above normal handling. Informed consent for the data collection was obtained from the horse owners prior to the study. Data from the same experiment have been used in two previous studies, investigating repeatability of vertical movement asymmetry parameters^25^ and repeatability of ROM for back and pelvic angles^26^, respectively.

### Markers

Spherical soft markers of 25 mm diameter were attached with double-adhesive tape. Three markers on a strip were placed; on the forehead (the lowest marker was used in the further analysis), on the withers (one on the highest point, two markers 20 cm lateral on each side, the central marker was used) and on the pelvis (a T-shaped strip fitted to the tubera sacrale and the craniodorsal aspects of both tubera coxae). Single markers were placed on the dorsal spinous processes of the thoracolumbar vertebrae T12, T15, T18, L3 and L5, and the sacrum (S5). Marker placement is visualised in Supplementary Fig. S1. To enable placing of markers on the same location each day, hair was clipped from a small area at marker locations.

### Data collection

Optical motion capture data were generated by Qualisys Motion Capture software (QTM^a^ version: 2.14, build: 3180). The measuring volume was covered by 28 high-speed infrared cameras (Oqus 700+^a^) set to a sampling frequency of 100 Hz. The total covered area was approximately 250 m^2^, height covered was at least 5 m. Calibration was done daily before the first measurement. The average calibration residual was 3.2 mm. A regular (25Hz) video camera (Sony HDR-CX330) was used to obtain synchronised video footage.

Kinematic registrations were made of the horses trotting in an indoor arena on a soft (sand and synthetic fibre) surface on the straight, and on left and right circles. Each trial included three measurements: straight line in hand (2 × 30 m) and lungeing on the left and then the right circle (each 25 s of data collection). This was done five times daily, on two consecutive days, totalling to 30 measurements per horse, 10 per path. Horses were also measured in straight line trot on hard (tarmac) surface and measurements were repeated twice on a third day after 30–55 days^25^, but these data were not included in the current study. An experienced equine veterinarian examined the horses on the day before the first measurement, found no clinical signs suggestive of back pain, and graded them as sound (‘fit to compete’) defined as less than 1 on a adapted version of the AAEP 0 to 5 lameness scale^27^, after evaluating them in walk and trot on the straight, on soft surface. The lameness grades were defined as previously described^28^: e.g. grade 0 (no lameness), and grade 1 (slight lameness in trot only). Half-grades were given if the lameness was perceived to be in-between grades. This lameness scale was routinely used by all veterinarians at the clinic where the study took place.

On each day, horses were first hand-walked for 5 minutes and lunged for 10 minutes as warm-up. Thereafter, markers were placed, always by the same researcher (AH). Trials were then performed with five-minute intervals between the first two trials and ten-minute intervals between the following trials. Circle radius was approximately 5 m (length of lunge-line standardized by a knot). Daily harrowing of the surface was done prior to the measurements. Measurements took place at each horse’s preferred speed and care was taken to maintain this speed consistently both within and between trials. Two handlers handled the horses, and each horse was always handled by the same handler.

In connection with each measurement, the optical motion capture data were manually inspected. Measurements with poor marker tracking or insufficient number of collected strides (< 5 strides) were discarded.

### Data analysis

Kinematic data were analysed using custom-written Matlab scripts. Stride segmentation was done based on the vertical maxima for the tubera sacrale marker, and pelvis roll to determine left vs right hind limb stance^29^. For this purpose, data were filtered using a zero-lag Butterworth high-pass filter with a cut-off frequency of 70% of the stride frequency^30^.

**Figure 1:**
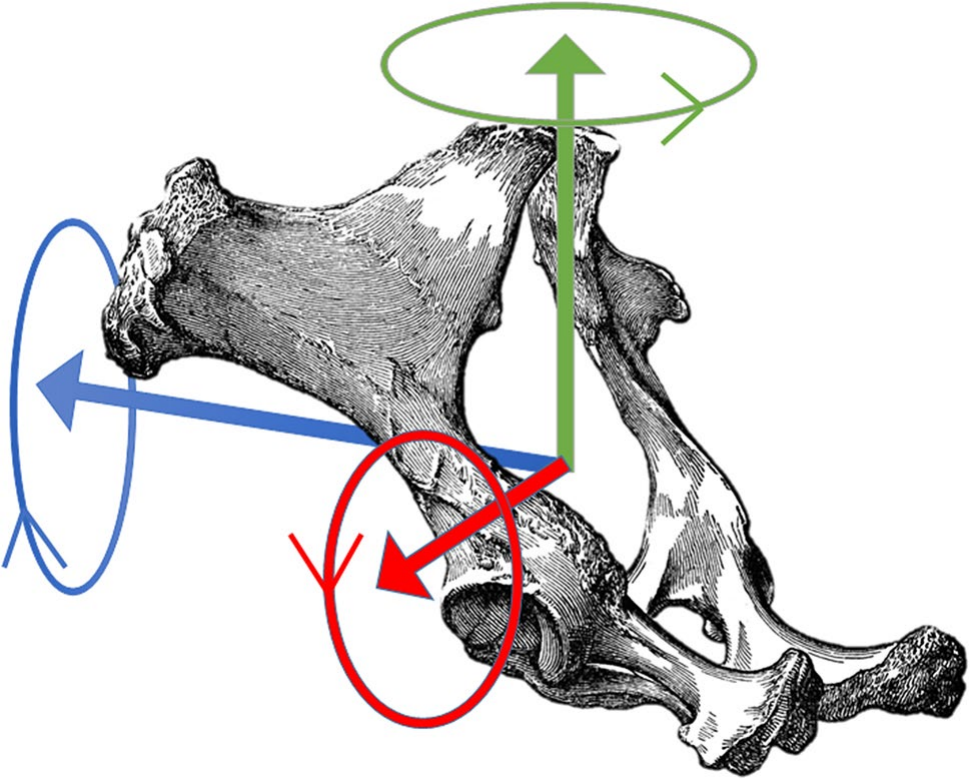
Illustration of pelvic rotations, roll (blue), pitch (red) and yaw (green). Cranial is to the left (blue arrow).

**Figure 2:**
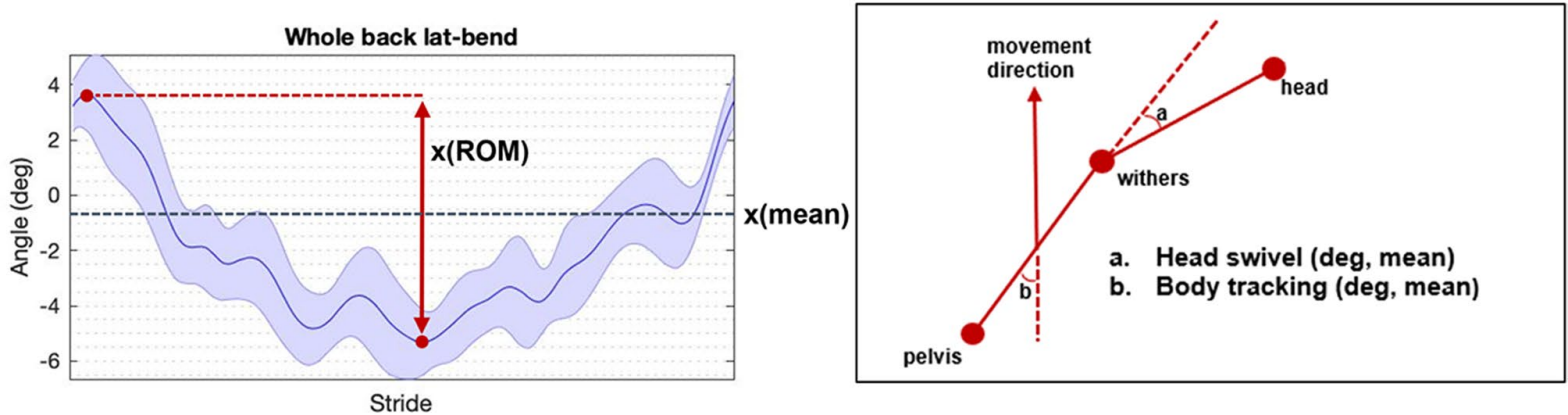
The left graph illustrates how stride mean and range of motion (ROM) were calculated, shown on an example of a lateral bending stride curve (blue line indicates the mean, shaded area the standard deviation). The right graph illustrates the variables **a** ‘Head swivel’ and **b** ‘Body tracking’, with angles indicated on the side where they are positive, e.g. head placed to the right of the long axis of the body.

From stride-segmented (unfiltered) optical motion capture data, the following variables were calculated: Flexion-extension and lateral bending angles for the whole (thoracolumbar) back were determined between the markers at the highest point of the withers, T15 and tubera sacrale, in the vertical plane (‘seen from the horse’s side’) for flexion-extension and in the horizontal plane (‘seen from above’) for lateral bending. Additionally, angles between each set of three markers (withers—T12-T15; T12-T15-T18; etc., as listed in Table 1) were calculated in the same planes, to represent flexion-extension and lateral bending of the back segments. Flexion-extension angles were defined as zero if the three markers were level, positive for flexion of the back and negative for extension. Lateral bending angles were defined as zero when markers were aligned in the sagittal plane, positive for bending of the back to the right and negative for bending to the left. Supplementary Fig S2 illustrates the back angle calculations, showing the withers—T12-T15 flexion-extension angle as an example. Pelvis roll (axial rotation) was determined relative to the horizontal, using the two lateral (tuber coxae) pelvic markers. Pelvis pitch (rotation in the vertical plane) was calculated using the marker at the tubera sacrale and the average of the two lateral (tuber coxae) pelvic markers, and pelvis yaw (rotation in the horizontal plane) was calculated using the two lateral pelvic markers; both were expressed relative to a line between the withers and tubera sacrale markers. Pelvic rotations, including positive direction of these, are illustrated in Fig. 1. Stride range of motion and stride mean (illustrated in Fig. 2) were calculated for each stride for the above-mentioned variables. Stride mean pelvis roll was used to approximate body lean of the horse^4^, and the vertical and horizontal planes were adjusted accordingly (tilted as much as the horse was leaning, based on the average in a moving window with the length of stride duration times sampling rate and centred on the data frame in question). This was done to avoid projection errors in (crosstalk between) lateral bending and flexion-extension angles by aligning the reference frame to the anatomical planes.

**Table 1:** Least square means estimates (Est) and standard errors (SE) by path, and estimated differences between paths including P-values (P), for stride mean flexion–extension (FE) and lateral bending (LB) variables (all in degrees).

Independent variables	Straight		Left		Right				
	Est	SE	Est	SE	Est	SE			
FE withers-T15-tuber sacrale	− 17.6	0.64	− 17.7	0.64	− 17.7	0.64			
FE withers-T12-T15	− 15.3	0.74	− 15.3	0.74	− 15.3	0.74			
FE T12-T15-T18	− 3.7	0.50	− 3.7	0.50	− 3.7	0.50			
FE T15-T18-L3	− 3.5	0.34	− 3.5	0.34	− 3.5	0.34			
FE T18-L3-L5	− 0.3	0.66	− 0.4	0.66	− 0.5	0.66			
FE L3-L5-tuber sacrale	0.4	0.81	0.3	0.81	0.3	0.81			
FE L5-tuber sacrale-S5	21.5	0.78	20.7	0.78	20.9	0.78			
LB withers-T15-tuber sacrale	− 0.1	0.42	− 3.9	0.42	3.5	0.42			
LB withers-T12-T15	− 0.7	0.69	− 2.1	0.68	0.8	0.68			
LB T12-T15-T18	− 0.2	0.67	− 1.3	0.67	0.8	0.67			
LB T15-T18-L3	1.7	0.60	0.6	0.60	2.7	0.60			
LB T18-L3-L5	− 0.1	0.46	− 1.2	0.46	0.9	0.46			
LB L3-L5-tuber sacrale	− 1.9	0.78	− 2.2	0.78	− 1.7	0.78			
LB L5-tuber sacrale-S5	2.9	0.73	2.7	0.73	3.2	0.73			
Pelvis roll	− 1.0	0.35	− 13.4	0.35	11.7	0.35			
Pelvis pitch	36.9	1.27	37.6	1.27	37.9	1.27			
Pelvis yaw	2.2	0.56	− 0.2	0.56	4.5	0.56			
Head swivel	− 1.5	0.87	− 6.7	0.85	3.3	0.85			
Body tracking	0.1	0.33	2.4	0.33	− 2.5	0.33			

	Straight-left			Straight-right			Left–right		
	Est	SE	P	Est	SE	P	Est	SE	P
FE withers-T15-tuber sacrale	0.06	0.03	0.07	0.10	0.03	0.004	0.04	0.02	0.07
FE withers-T12-T15	− 0.02	0.04	0.77	0.01	0.04	0.77	0.04	0.03	0.71
FE T12-T15-T18	− 0.02	0.01	0.07	− 0.06	0.01	< 0.0001	− 0.04	0.01	< 0.0001
FE T15-T18-L3	0.05	0.01	< 0.0001	0.04	0.01	< 0.0001	− 0.01	0.01	0.12
FE T18-L3-L5	0.09	0.01	< 0.0001	0.16	0.01	< 0.0001	0.08	0.01	< 0.0001
FE L3-L5-tuber sacrale	0.05	0.01	< 0.0001	0.07	0.01	< 0.0001	0.02	0.01	0.001
FE L5-tuber sacrale-S5	0.82	0.03	< 0.0001	0.64	0.03	< 0.0001	− 0.18	0.02	< 0.0001
LB withers-T15-tuber sacrale	3.75	0.05	< 0.0001	− 3.61	0.04	< 0.0001	− 7.37	0.03	< 0.0001
LB withers-T12-T15	1.39	0.04	< 0.0001	− 1.48	0.04	< 0.0001	− 2.87	0.03	< 0.0001
LB T12-T15-T18	1.08	0.02	< 0.0001	− 1.05	0.02	< 0.0001	− 2.13	0.01	< 0.0001
LB T15-T18-L3	1.11	0.01	< 0.0001	− 0.99	0.01	< 0.0001	− 2.10	0.01	< 0.0001
LB T18-L3-L5	1.10	0.02	< 0.0001	− 1.05	0.02	< 0.0001	− 2.15	0.01	< 0.0001
LB L3-L5-tuber sacrale	0.34	0.01	< 0.0001	− 0.22	0.01	< 0.0001	− 0.56	0.01	< 0.0001
LB L5-tuber sacrale-S5	0.22	0.01	< 0.0001	− 0.24	0.01	< 0.0001	− 0.45	0.01	< 0.0001
Pelvis roll	12.36	0.07	< 0.0001	− 12.72	0.07	< 0.0001	− 25.08	0.05	< 0.0001
Pelvis pitch	− 0.67	0.03	< 0.0001	− 0.95	0.03	< 0.0001	− 0.29	0.02	< 0.0001
Pelvis yaw	2.46	0.03	< 0.0001	− 2.22	0.03	< 0.0001	− 4.68	0.02	< 0.0001
Head swivel	5.23	0.29	< 0.0001	− 4.80	0.29	< 0.0001	− 10.03	0.21	< 0.0001
Body tracking	− 2.30	0.08	< 0.0001	2.62	0.08	< 0.0001	4.92	0.05	< 0.0001

Each model also contained a speed*path fixed effect (supplement 1) and included between 8055 and 8073 observations (strides) from 12 horses.

Speed was determined from the movement of the tubera sacrale marker in the horizontal plane. Body tracking, i.e. the orientation of the horse’s body (a line between the withers and tubera sacrale) relative to the direction of movement (determined from the speed vector), was calculated. Cervical lateral bending (head swivel) was calculated as the angle between the body and the neck (a line between the head marker and the withers marker). These angles are illustrated in Fig. 2. Head swivel is positive if the head is to the right of the body axis and body tracking angle is positive for ‘forehand to the right’—‘hind quarters to the left’ deviation.

### Statistical analysis

Kinematic variables were scrutinised, both measurement time-series and stride-segmented data. Boxplots of raw data and scatterplots of measurement mean data were used for description.

Mixed models were constructed (R version 3.6.1) to study differences between paths for back and pelvic kinematics, body tracking and head swivel. Speed was included in all models regardless if significant, to control for minor speed variations between measurements. Random effect in all models was measurement within horse. Alpha was set to 0.05 in all analyses. All presented models were made from stride-by-stride data. Both ROM and stride means were analysed for back and pelvic variables, whereas for head swivel and body tracking and speed only stride means were analysed. R packages that were used included: lme4, lmerTest and emmeans.

Before analysis, variables were plotted in quantile-quantile plots. Variables that did clearly not conform to normality were transformed along the ladder of powers to find the optimal transformation. These variables (ROM for withers-T12-T15 lateral bending and flexion-extension, ROM for T15-T18-L3 lateral bending and ROM for pelvis roll) were then analysed both transformed and untransformed. As the conclusions from the models were similar in both transformed and untransformed formats, the untransformed versions were used. Two categories of models were made, addressing the first and the second hypothesis of the study, respectively:

Comparison between straight line and circles: these models included mean and ROM for back angles and pelvic rotations as dependent variables, and path and speed and their interaction as fixed effects. Least square means for the three different paths were evaluated at speed grand mean (across all horses and measurements). Multiple comparisons of least square means within each model were adjusted for using the false discovery rate method (in emmeans).

Correlations between back variables and the other studied variables: these models included stride mean and ROM for whole back lateral bending and flexion-extension as dependent variables. The independent variables were pelvic rotations, speed, head swivel and body tracking, and speed. In general, mean and ROM variables were evaluated for mean and ROM dependent variables, respectively, with the exception of speed, and stride mean head swivel and body tracking, which were entered into all models. When flexion-extension mean or ROM was modelled, head swivel and body tracking were entered as absolute values. All tested variables were plotted versus the dependent variable in order to rule out modality or exponential relationships. These models were made separately for each path, one for straight line data, one for left circle data, and so on. This was in order to verify or refute consistency over analyses. During preliminary analysis, using the Akaike information criterion, it was found that essentially all independent variables contributed in these models. Because of this, all fixed effects were kept, and no model reduction was made.

## Results

**Figure 3:**
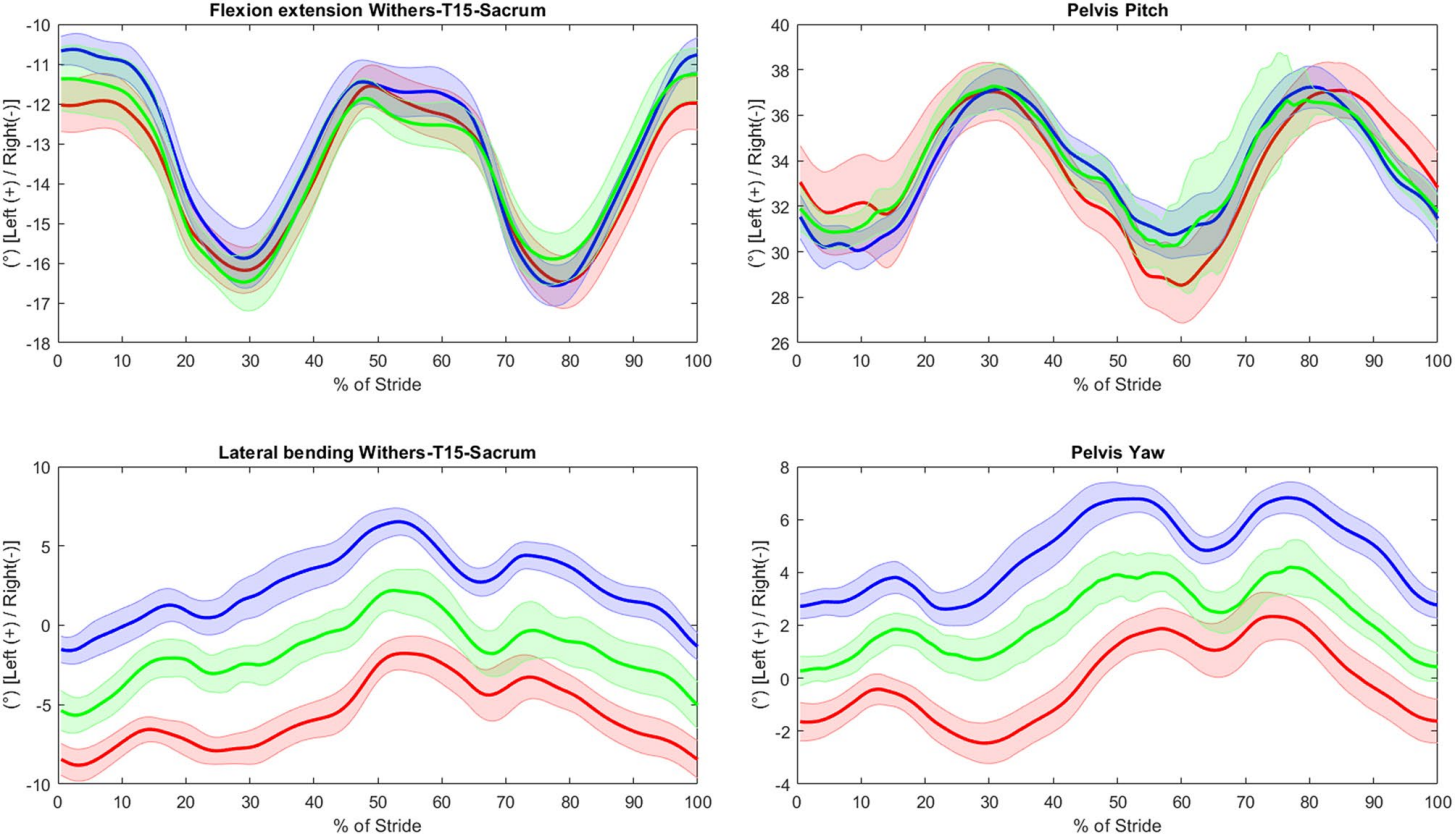
Stride curves (normalized to 0–100% of the stride) for whole back flexion–extension and lateral bending, and pelvis pitch and yaw, for one horse (horse 12). Lines indicate the mean and shaded area the standard deviation, for left circle (red), right circle (blue) and straight line (green).

**Figure 4:**
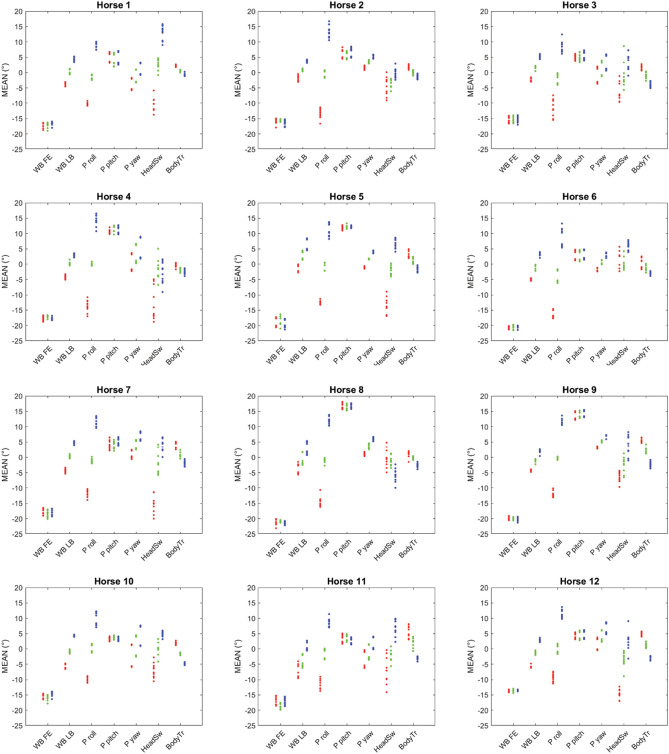
Measurement means for stride mean for whole back (WB) flexion–extension (FE) and lateral bending (LB), pelvis (P) roll, pitch and yaw, head swivel and body tracking. The plots contain data from 12 horses on three paths (red = left, blue = right, green = straight line), studied during two different days for five occasions each day (n = 355 measurements in total).

**Figure 5:**
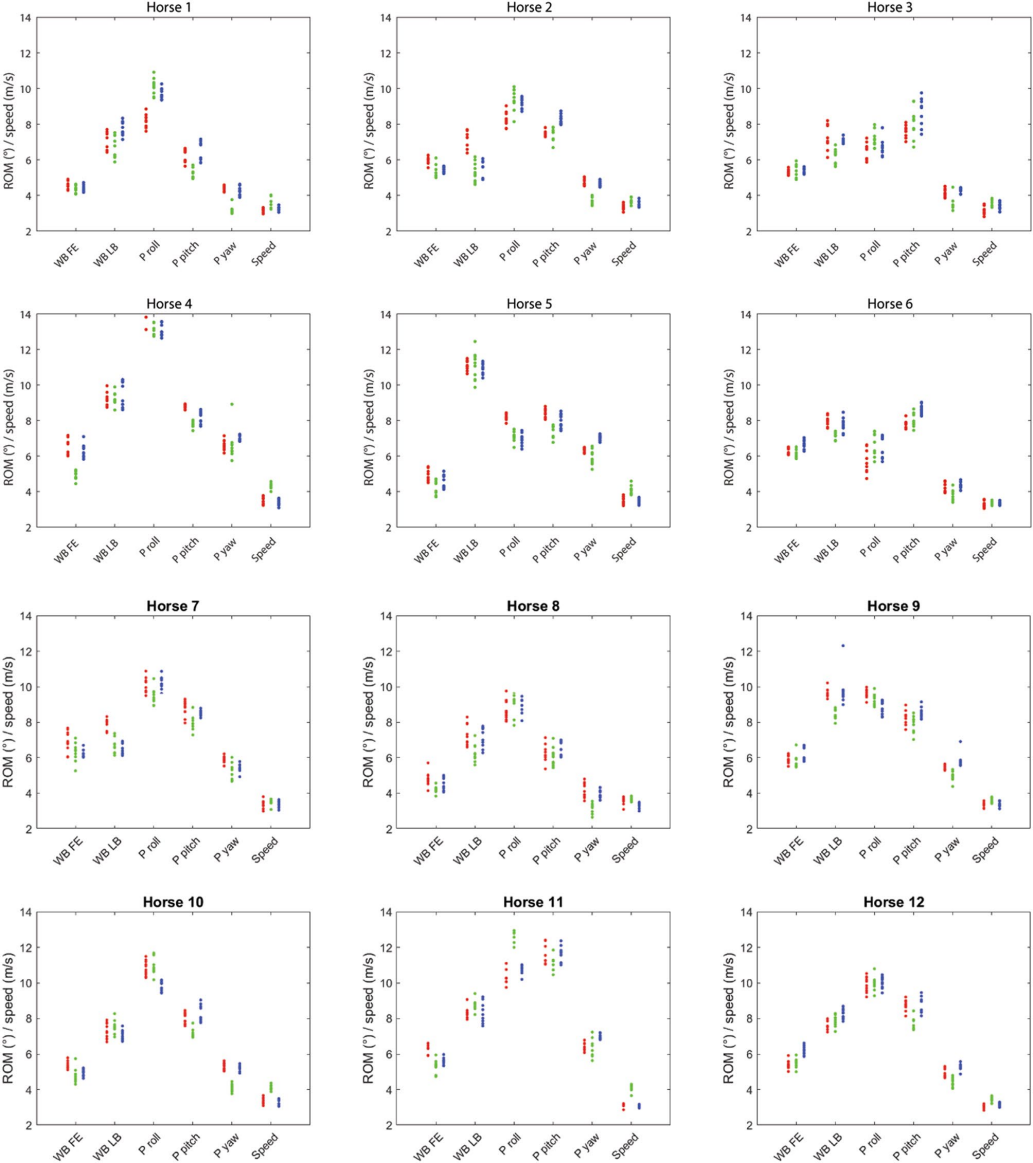
Measurement mean data plotted by horse for selected stride range of motion (ROM) variables for whole back (WB) flexion–extension (FE) and lateral bending (LB), pelvis (P) roll, pitch and yaw, and speed. The plots contain data from 12 horses on three paths (red = left, blue = right, green = straight line), studied during two different days for five occasions each day (n = 355 measurements in total).

**Figure 6:**
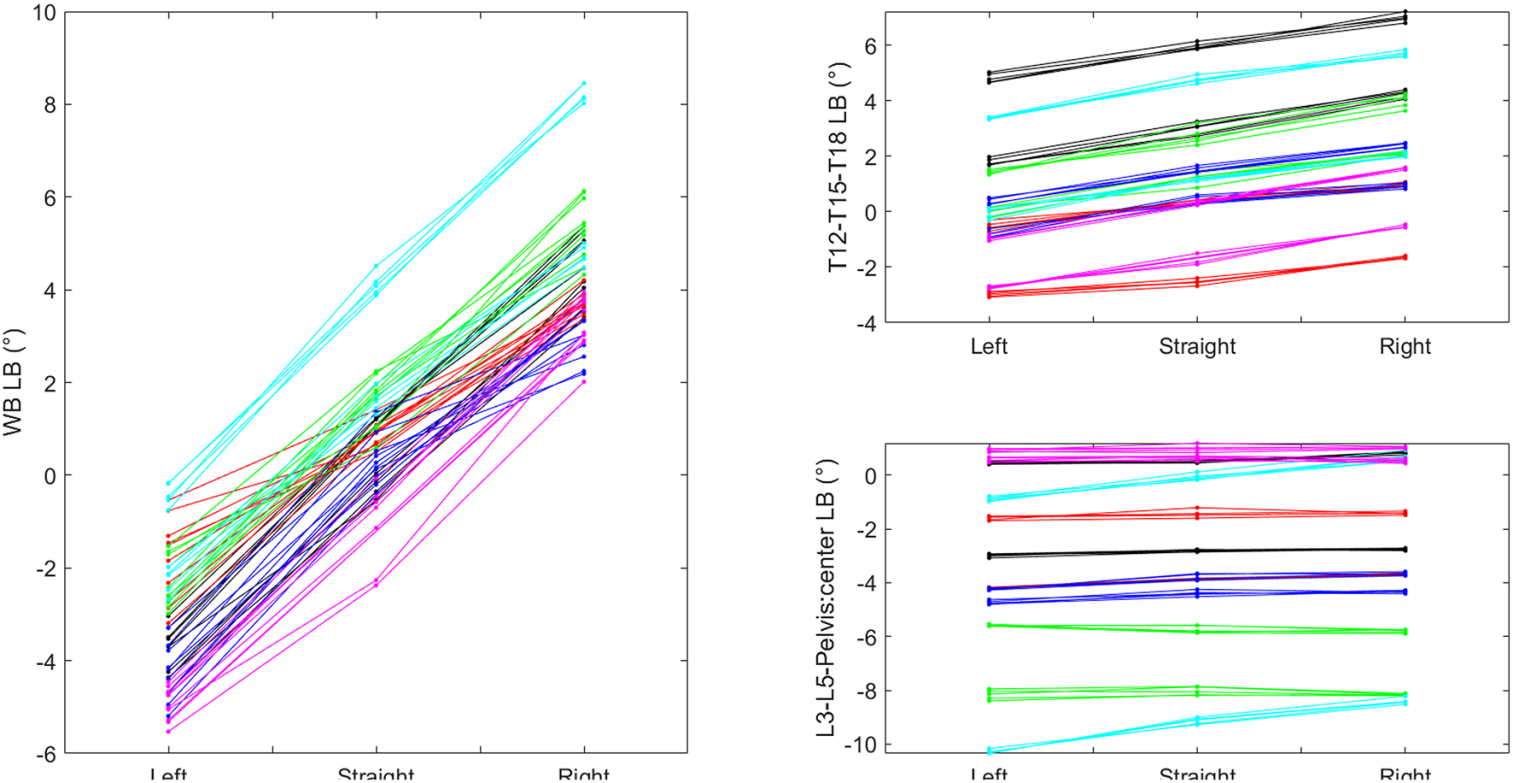
Mean values per measurement for whole back (WB) stride mean lateral bending (LB) for selected segment angles in 6 horses (10 trials per horse) of the 12 horses included in the study. Each horse is represented by a colour.

The 12 horses contributed 8073 strides in total to the dataset (with 1338/3270/3465 strides respectively, from the straight line and left/right circles). The strides emanated from a total of 355 measurements across the 12 horses. 5 of the 360 measurements obtained had to be discarded, leaving a minimum of eight measurements per horse and path (two horses lost one straight-line measurement each and one horse lost two straight-line measurements and one measurement on the left circle). The number of strides per measurement included in the statistical analyses varied between 4 and 20 for straight line (median 11), left circle 6–42 (median 28) and right circle 18–41 (median 29). Speed was higher on a straight line, 3.73 (SE 0.04) m/s compared to on left/right circles, 3.31 (SE 0.04) m/s and 3.34 (SE 0.04) m/s, and left and right circles also differed significantly (p < 0.0001 for the three two-way comparisons). Across all horses, circle radius varied between 4.8 and 5.2 m for the left circle and for the right circle between 4.8 and 5.3 m. Examples of stride curves for analysed variables are displayed in Fig. 3. Individual variation is illustrated in Figs. 4, 5 and 6.

Table 1 shows least square stride mean values and the estimated differences between straight line and left and right circle. Table 2 shows stride ROM and the differences in ROM between straight line, left circle and right circle. In both tables, a positive difference indicates a smaller value for circle compared to straight line. There were significant differences between straight line and left/right circles for most variables, and between left and right circles for many variables. Estimated differences were generally small. Estimates for the speed-path interaction can be found in Supplementary Table S1.

**Table 2:** Least square means estimates (Est) and standard errors (SE) by path, and estimated differences between paths including P-values (P), for stride range of motion (ROM) flexion–extension (FE) and lateral bending (LB) variables (all in degrees).

Independent variables	Straight		Left		Right				
	Est	SE	Est	SE	Est	SE			
FE withers-T15-tuber sacrale	5.4	0.19	5.6	0.19	5.5	0.19			
FE withers-T12-T15	4.3	0.40	4.3	0.40	4.3	0.40			
FE T12-T15-T18	2.6	0.22	2.3	0.22	2.3	0.22			
FE T15-T18-L3	2.5	0.10	2.3	0.10	2.6	0.10			
FE T18-L3-L5	2.6	0.14	2.5	0.14	2.5	0.14			
FE L3-L5-tuber sacrale	2.7	0.23	2.8	0.23	2.8	0.23			
FE L5-tuber sacrale-S5	3.6	0.12	3.8	0.12	3.7	0.12			
LB withers-T15-tuber sacrale	7.4	0.37	8.2	0.37	8.0	0.37			
LB withers-T12-T15	8.2	1.00	8.8	1.00	8.6	1.00			
LB T12-T15-T18	4.9	0.50	5.3	0.50	5.0	0.50			
LB T15-T18-L3	3.8	0.50	4.1	0.50	4.0	0.50			
LB T18-L3-L5	4.0	0.26	4.3	0.26	4.4	0.26			
LB L3-L5-tuber sacrale	4.3	0.34	4.5	0.34	4.8	0.34			
LB L5-tuber sacrale-S5	4.7	0.24	4.9	0.24	4.8	0.24			
Pelvis roll	9.5	0.56	9.4	0.56	9.3	0.56			
Pelvis pitch	7.8	0.36	8.1	0.36	8.3	0.36			
Pelvis yaw	4.6	0.30	5.2	0.30	5.3	0.30			

	Straight-left			Straight-right			Left–right		
	Est	SE	P	Est	SE	P	Est	SE	P
FE withers-T15-tuber sacrale	− 0.18	0.025	< 0.0001	− 0.03	0.025	0.25	0.15	0.018	< 0.0001
FE withers-T12-T15	− 0.05	0.027	0.13	− 0.01	0.027	0.84	0.04	0.019	0.09
FE T12-T15-T18	0.22	0.018	< 0.0001	0.23	0.018	< 0.0001	0.01	0.013	0.65
FE T15-T18-L3	0.25	0.018	< 0.0001	− 0.09	0.018	< 0.0001	− 0.34	0.013	< 0.0001
FE T18-L3-L5	0.05	0.016	0.006	0.05	0.016	0.006	0.00	0.012	0.87
FE L3-L5-tuber sacrale	− 0.07	0.018	0.0003	− 0.09	0.018	< 0.0001	− 0.02	0.013	0.05
FE L5-tuber sacrale-S5	− 0.22	0.023	< 0.0001	− 0.08	0.023	0.001	0.15	0.016	< 0.0001
LB withers-T15-tuber sacrale	− 0.87	0.038	< 0.0001	− 0.62	0.037	< 0.0001	0.25	0.027	< 0.0001
LB withers-T12-T15	− 0.65	0.045	< 0.0001	− 0.42	0.045	< 0.0001	0.23	0.032	< 0.0001
LB T12-T15-T18	− 0.38	0.038	< 0.0001	− 0.15	0.037	0.0001	0.23	0.027	< 0.0001
LB T15-T18-L3	− 0.36	0.028	< 0.0001	− 0.25	0.027	< 0.0001	0.11	0.020	< 0.0001
LB T18-L3-L5	− 0.27	0.028	< 0.0001	− 0.34	0.027	< 0.0001	− 0.07	0.020	0.0004
LB L3-L5-tuber sacrale	− 0.21	0.025	< 0.0001	− 0.50	0.025	< 0.0001	− 0.28	0.018	< 0.0001
LB L5-tuber sacrale-S5	− 0.20	0.026	< 0.0001	− 0.11	0.026	< 0.0001	0.09	0.018	< 0.0001
Pelvis roll	0.14	0.065	0.05	0.15	0.064	0.05	0.01	0.046	0.87
Pelvis pitch	− 0.31	0.032	< 0.0001	− 0.53	0.032	< 0.0001	− 0.21	0.023	< 0.0001
Pelvis yaw	− 0.53	0.027	< 0.0001	− 0.69	0.026	< 0.0001	− 0.16	0.019	< 0.0001

Each model also contained a speed*path fixed effect (supplement 1) and included between 8055 and 8073 observations (strides) from 12 horses.

### Straight-line movement

For stride mean flexion-extension (Table 1), the whole back angle (between withers, T15 and the centre point between the two tubera sacrale) on the straight line was − 17.6° (minus sign indicating extension, i.e. T15 is below withers and sacrum). The least square means for the segment angles range from extension in the cranial thoracic back (− 15.3°, illustrated in Supplementary Fig S2) towards neutral (horizontal alignment of the markers) at the L3 segment (− 0.34°), to a slight flexion at L5 (0.40°) and flexion at the lumbosacral junction (21.5°). Flexion-extension ROM for the whole back (Table 2) was 5.42° on the straight line. The highest ROM for flexion-extension of the back segments was found at T12 (4.28°).

Stride mean lateral bending (Table 1) on the straight line ranged between − 1.90° and 2.93° for the segments and the whole back. These figures must be interpreted with caution, due to possible slight off-midline placement of markers (cf. Fig. 5). Lateral bending ROM for the whole back was 7.35°. For the segments, the highest ROM was found for the T12 segment (8.18°). Across all horses, cervical lateral bending (head swivel) was slightly to the left on the straight line (least square mean − 1.50°). Stride mean pelvis roll (body lean) was almost zero. Pelvis pitch least square mean was 36.9° (indicating an upward slope from the midpoint between the two tubera coxae to the tuber sacrale). Stride mean pelvis yaw showed positive rotation (‘tail towards the right’) on the straight line (2.24°). Ranges of motion for pelvis roll, pitch and yaw were 9.49°, 7.77° and 4.64° (Table 2).

The body tracking angle indicated slight tracking of the hindquarters to the right (or forehand to the left) on the straight (0.13°). Combining all results related to horse straightness, the average horse of the study group showed left cervical bending, tracking of the hindquarters to the right, and ‘tail to the right’ yaw rotation of the pelvis relative to the long axis of the body on the straight line.

### Changes in movements on the circle compared to a straight line

Stride mean flexion-extension (Table 1) indicates that the whole back was slightly more extended on the circle compared to the straight line (estimated differences comparing to straight: left 0.06°; right 0.10°). The same was true for the segments except for T15; statistically significant but small differences were found, with the largest differences at the lumbosacral junction (left 0.82° right 0.64°).

For stride mean lateral bending, the whole back (estimated differences left 3.75°; right − 3.61°), as well as all segments, showed left bending on the left circle and right bending on the right circle. The contribution from each of the segments to the whole back lateral bending was approximately equal over the more cranial segments, T12-L3 (estimated differences left 1.1° to 1.4°; right − 1.5° to − 1.0°, compared to straight). For the most caudal segments (L5 and sacral segments), differences were smaller (left 0.22° to 0.34°; right − 0.24° to − 0.22°, compared to straight). Cervical lateral bending to the inside of the circle was symmetric compared to the straight line for left and right circles (differences; left 5.23°; right − 4.80°), but since horses showed left lateral bending on the straight, least square means suggest relatively more bending to the inside on the left circle (least square means − 6.73° vs. 3.30°). Body tracking showed about the same estimated ‘forehand in—hindquarters out’ deviation with hindquarters towards the left on the left circle and to the right on the right circle (differences left − 2.30°; right 2.62°, compared to straight).

Differences between circle and straight in ROM were small. For flexion-extension there was no consistent direction of change (Table 2), but estimate signs are generally consistent for the same angle/segment between left and right circles. For lateral bending significant differences indicated increased ROM on the circle. For the whole back the estimated differences were − 0.87° and − 0.62° compared to the straight (negative sign indicates higher values on the circles).

Stride mean pelvis roll showed approximately the same estimated differences, with roll to the left on the left circle, and to the right on the right circle (12.4° and 12.7°), compared to almost zero degrees on the straight line (Table 1). For pelvis pitch, differences to straight were − 0.67° and − 0.95°, indicating slightly more extension. Stride mean pelvis yaw was (tail) to the inside compared to straight, to the right on the right circle (difference − 2.22°) and to the left on the left circle (difference 2.46°). Pelvis roll ROM decreased slightly on the circle, by 0.14°–0.15°, whereas pelvis pitch and yaw ROM increased between − 0.31° and − 0.69° (Table 2).

### Relationships between back movements and other variables

Whole back lateral bending stride mean showed the strongest correlation to stride mean pelvis yaw (Supplementary Table S2). Pelvis yaw estimates ranged between 1.1° to 1.3° across all three paths, which suggests that each degree of increased yaw to one side is associated with slightly more than 1° increase in lateral bending to the same side. All other variables tested (stride mean pelvis roll and pitch, head swivel, body tracking and speed) were statistically significant, except for mean pelvis pitch for the left circle (p = 0.46) but estimates for variables other than speed were below 0.1°. Estimates for these latter variables, including speed, remained small or proved unstable when entering or removing variables in the models. Accordingly, except for pelvis yaw, estimates should be interpreted with caution. For the remaining whole back variables (lateral bending ROM and flexion-extension stride mean and ROM), model estimates proved to be generally unstable when adding or removing (independent) variables, and/or were inconsistent between left and right circles (Supplementary Table S2). A possible reason is that only stride mean lateral bending showed substantial difference between circle and straight line. Scatterplots of whole back variables vs. the variable with the strongest association in each model are found in Supplementary Fig. S3.

## Discussion

The current study explores adaptations in horse body movements when trotting in circles. In line with the hypothesis, the current study found significant differences between circles and straight line for most of the studied variables. The findings included lateral bending of the neck and thoracolumbar back to the inside of the circle, along with yaw of the pelvis turning the tail towards the inside on the circles. These changes are towards alignment between the horse’s vertical column and the circular track, in accordance with equestrian theory. However, all differences found were small and may or may not be visible for a human observer. Cervical lateral bending in the direction of motion (left − 5.23°; right + 4.80° vs. straight) is likely to be visible. Lateral bending of the back (≤ 3.6°–3.8°) was small for each segment but may be visible for the back as a whole. For stride mean pelvis yaw, the estimated differences compared to straight were also small (left 2.46°; right − 2.22° vs. straight). In equestrian literature, lateral bending of the trunk is often referred to and is a core part of the training goals, even though there are some equestrian authors that dispute the horse’s ability to bend laterally other than in the cervical spine^31^. It is unclear whether these observations that are reported in equestrian literature, are based on actual lateral bending of the back, or reflect the concurrent changes in limb movement^1^ and muscle activity^32^ when moving in circles, e.g. shorter stride length for inside limbs compared to outside limbs. The slight increase in extension of the back and differences in back and pelvic ROM are unlikely to be appreciable with an unaided eye and can be argued to be too small to be biologically meaningful as single findings. The increase in body lean found on the circle compared to straight line is likely to be visible (12°–13°, Table 1).

Previous studies on back movement in trot include treadmill studies using optical motion capture^20–22^, as well as overground studies using an inertial measurement unit (IMU) set-up that has been validated against optical motion capture^33^. These studies have quantified thoracolumbar segment angles relative to the room; angles equivalent to roll, pitch, and yaw, but were referred to as axial rotation, flexion-extension, and lateral bending, respectively. This means that those studies looked at the amount of rotation of a given anatomical point, whereas the present study quantified angles spanned by three different anatomical points, rendering the results only approximately comparable. Back ROM has been compared between straight line and circle using IMUs^5,24^. It was found that ROM generally increased on the circle. For flexion-extension increases were 1° to 2°, and for lateral bending ROM increases of about 20° were seen on the circle^5^. The increases in lateral bending likely reflect the turning motion of the horse during each stride, rather than changes in movement within the back. In the present study, increases in lateral bending ROM were much smaller (left: 0.87° and right: 0.62°), though still significant. Comparing the results for straight line back ROM in the current study to previous treadmill studies^20–22^, those studies found slightly higher ROM, but despite differences in how angles were calculated the relative distribution over different anatomical locations was similar: for both flexion-extension and lateral bending the highest values were found for the most cranial segments, decreasing towards the thoracolumbar junction and slightly increasing again towards the lumbosacral joint.

Comparing the results of the current study to cadaver studies, the movement contributions of the different segments look quite different for lateral bending, but more similar for flexion extension. Flexion-extension ROM was found to be highest between T17 and L1 (thoracolumbar junction) and to decreased both cranially and caudally, while still remaining fairly similar to the most moveable segment, 2°–4° of movement between adjacent vertebrae^34,35^. This is in line with the current results of 3°–4° over several vertebrae. Lateral bending ROM was found to be greatest in the caudal half of the thoracic spine (maximum 11° at T11) and to decreased towards the lumbar spine (on average 3°)^35^. This can be attributed to the sagittal orientation of the articular facets and the intertransverse joints^36^. In the current study, ROM was relatively similar between the thoracic and lumbar back, 4°–8° for individual segments. For the thoracic back, this is considerably lower compared to the cadaveric study^35^, considering that we measured the combined movement over several vertebrae. It is plausible that the horse did not use its maximum mobility of the back to trot around the 10 m circle. The relatively higher ROM for the lumbar back in this study may to some extent be related to projection errors originating from axial rotation of the back during the stride^37^. A previous study that validated thoracolumbar segment angles derived from skin markers against bone-fixated markers using optical motion capture reported axial rotation to interfere only with lateral bending, not flexion-extension angles, and only in the caudal thoracic and cranial lumbar areas. It was though concluded that these errors are practically negligible for trot^37^, angles for all thoracolumbar segments were deemed valid. In that study, data collection was performed on a treadmill, i.e. in straight line. In the current study the reference frame for the thoracolumbar angles was corrected for horse body lean, aiming to ensure equivalent calculations for straight line and circles.

Contrary to our hypothesis, changes in flexion-extension and lateral bending of the thoracolumbar back between straight line and circles were not strongly correlated to changes in pelvic rotations, cervical lateral bending, body tracking or body lean. The only consistent correlation found was between stride mean pelvis yaw and lateral bending. This relationship suggests that the thoracolumbar and lumbosacral back accommodate to the bend of the circle in a coordinated manner. In the present study, body lean (stride mean pelvis roll) had only a weak association with the degree of lateral bending to the inside of the circle. This would suggest that increased lateral bending may not be a reason why well-ridden horses lean less into the circle^5^. On the other hand, an association may still be present within the same horse over a period of schooling.

In this group of 12 horses, there were several statistically significant, but small, differences between left and right circles. The clinical or biological significance of these is uncertain. A number of previous studies have found various differences between left and right circles: In one study, horses leaned less than expected on right circles, and more than expected on left circles^4^. In another study, left-right differences in head and pelvis vertical maxima increased significantly with speed on the left but not the right circle in a group of sound to mildly lame horses^38^. Differences in absolute vertical movement symmetry have also been found^7,10^. These findings support that horses may adapt differently to moving on left versus right circles. Reasons for this can be laterality, painful conditions, training and/or habit^39^. However, study designs with non-randomised left-right order should also be considered. It would be interesting to investigate correlations between back movement asymmetries and vertical movement asymmetries. The results of the current study indicate that it would then be necessary to correct or control back angles for asymmetries in marker placement, e.g. analyse left-right circle differences. From marker-based angles alone it is impossible to know at what value the horse’s back movements are truly symmetrical, considering that in the current study markers were always placed by the same person and great care was taken in placing them correctly. Investigating links between movement asymmetries in various parts of the horse’s body is generally an interesting topic that warrants further study, preferably including larger groups of horses.

In the group of horses currently studied, individual variation in adaptations to circular movement, and individual variation in left-right differences are evident in Figs. 4, 5 and 6. Both the offset between days and non-zero values on the straight can reflect inconsistency or off-midline marker placement or anatomical asymmetries but may also be true asymmetries in the horse’s movements. For whole back stride mean lateral bending, all horses showed left bending on the left and right bending on the right circle, but some horses showed varying amounts of bending per trial/measurement (horse 5, 8 and 11), which causes overlap between circles and straight line (Fig. 4). A similar pattern is evident for pelvis yaw, e.g. for horse 3 and 10. Further, most horses showed an offset to the right in yaw (straight line/black dots above zero in Fig. 4). This is also evident from the least square means in Table 2. For pelvis roll, there is a clear difference between the movement directions for all horses. For cervical lateral bending, all horses except horse 6 and 8 showed left bending on the left circle and right on the right. Across trials, some horses showed more variation in cervical lateral bending than the others, e.g. horse 1. Overall, horses were consistent with regard to the difference between circle and straight, over both days, even if offsets between days can be clearly seen in some parameters (Figs. 4, 5, 6).

In a previous study using data from the same experiment, repeatability of ROM for back and pelvic angles, and for stride mean head swivel and body tracking, was investigated^26^. Repeatability was found to be good to fair for whole back and pelvic angles, but not as good for segment angles. The latter are more sensitive to small variations in marker placement, due to the short distances between markers. Another possible factor is measurement error. The average residual after interpolation of the position of the markers, when several cameras track the same marker, was 3.2 mm in both this and the previous study. At maximum, this corresponds to 1.2° error for a segment of the length 0.15 m (relevant for back segments) and 0.4° for a segment of the length 0.5 m (relevant for all other variables) for a single data frame. Since marker position estimation errors can be expected to be randomly distributed in both space and time, the effective angle error across a number of strides will be much smaller and likely less important compared to variations in marker placement. In the current study, there was considerable variation between horses and for stride mean angles also between the two measurement days, yet the overall trends for circle versus straight differences were comparably systematic and consistent. We therefore expect that the direction of changes will be consistent if the study would be repeated, whereas the exact figures should be interpreted with more caution, especially for the back segments.

A limitation of the study is the fairly small group of horses, but a benefit is that these horses were measured multiple times. The order of left and right circles was not randomised, but short intervals between repeats likely reduced the influence of left-right circle order. Skin markers are susceptible to skin displacement^40^. However, pairwise comparisons between conditions, e.g. straight lines and circles, are less affected by this problem. The reference frame for the thoracolumbar angles was corrected for horse body lean, but on a stride mean basis. This minimizes the risk of erroneous differences between straight line and circles. However, there is currently no validated method available to determine axial rotation of the thoracolumbar back accurately for each segment using skin markers, which would have been needed to eliminate this as a source of error. Horse owners were asked retrospectively to complete a questionnaire with questions based on descriptions of sidedness in equestrian literature. Unfortunately, comprehensive interpretation of the answers proved difficult, and we therefore refrained from further analysis. For future research, including walk and canter would be of great interest as those gaits are clinically important to assess spinal mobility, in addition to the trot. Besides, it would be interesting to repeat the study under tack. To overcome practical issues in marker placement in combination with a saddle and rider, this could be done with IMUs^19^. The current study evaluated circle-induced changes on whole-stride level, and further work is needed to understand back movements in greater temporal resolution, possibly combining several variables using machine learning methods, as is now being applied in human gait analysis^41^.

## Conclusion

Though this is considered a fact within equestrian dressage, the current study is the first to document that horses show increased lateral bending of the back to the inside when moving in circles. The same is true for the neck. Individual variation was evident, but each horse was generally consistent in its back motion pattern over multiple trials and consecutive days. This study adds to the biomechanical understanding of the equine back, knowledge that is useful in clinical assessment of back (dys)function. The addition of walk and canter and measurements under tack would complete this dataset and fill in further knowledge gaps within equine biomechanics and equestrianism.

## Supplementary Information

Supplementary Information 1.

## Data Availability

The datasets generated during and/or analysed during the current study are available in the Figshare repository, https://doi.org/10.6084/m9.figshare.14371718.
