# Supplementary material for: Differences in equine spinal kinematics between straight line and circle in trot
Source: Sci Rep. 2021 Jun 18;11:12832. doi: 10.1038/s41598-021-92272-2 (PMC8213771; doi:10.1038/s41598-021-92272-2)

Supplementary Information:

Differences in equine spinal kinematics between straight line and circle in trot

A. Byström, A.M. Hardeman, F.M. Serra Bragança, L. Roepstorff, J.H. Swagemakers, R. van Weeren, A. Egenvall

Supplementary Table S1.
Estimates for the speed-path interactions for models presented in Tables 1 (for stride mean values) and 2 (for ROMs- range of motion). Each model also included path as fixed effect, and is based on between 8055 and 8073 observations (strides) from 12 horses.

	Straight			Left			Right		
Variable - means	Est	SE	P	Est	SE	P	Est	SE	P
FE withers - T15 - tuber sacrale	-0.18	0.062	0.004	-1.06	0.072	<0.0001	-0.43	0.063	<0.0001
FE withers - T12 - T15	0.35	0.080	<0.0001	-0.49	0.092	<0.0001	0.23	0.082	0.01
FE T12 - T15 - T18	-0.03	0.019	0.08	-0.18	0.022	<0.0001	-0.12	0.019	<0.0001
FE T15 - T18 - L3	-0.30	0.015	<0.0001	-0.37	0.017	<0.0001	-0.32	0.015	<0.0001
FE T18 - L3 - L5	-0.21	0.017	<0.0001	-0.50	0.020	<0.0001	-0.39	0.017	<0.0001
FE L3 - L5- tuber sacrale	-0.08	0.018	<0.0001	0.00	0.021	0.93	-0.06	0.019	0.003
FE L5 - tuber sacrale - S5	-1.11	0.053	<0.0001	-1.75	0.061	<0.0001	-1.60	0.054	<0.0001
LB withers - T15 - tuber sacrale	0.04	0.086	0.64	-1.44	0.099	<0.0001	1.41	0.088	<0.0001
LB withers - T12 - T15	0.12	0.067	0.07	-0.45	0.078	<0.0001	1.31	0.069	<0.0001
LB T12 - T15 - T18	-0.01	0.031	0.74	-0.54	0.036	<0.0001	0.28	0.032	<0.0001
LB T15 - T18 - L3	-0.04	0.027	0.10	-0.52	0.031	<0.0001	0.48	0.028	<0.0001
LB T18 - L3 - L5	-0.02	0.030	0.61	-0.41	0.034	<0.0001	-0.11	0.030	0.0003
LB L3 - L5- tuber sacrale	0.02	0.027	0.56	-0.08	0.031	0.01	-0.23	0.028	<0.0001
LB L5 - tuber sacrale - S5	-0.04	0.021	0.04	0.11	0.024	<0.0001	-0.08	0.021	0.0001
Pelvis roll	-0.12	0.131	0.37	6.45	0.152	<0.0001	-7.20	0.134	<0.0001
Pelvis pitch	1.34	0.054	<0.0001	2.73	0.062	<0.0001	2.13	0.055	<0.0001
Pelvis yaw	0.02	0.050	0.73	-1.02	0.058	<0.0001	0.55	0.051	<0.0001
Head swivel	0.51	0.556	0.36	-1.83	0.633	0.004	8.47	0.562	<0.0001
Body tracking	-0.11	0.146	0.43	0.45	0.168	0.01	-1.28	0.149	<0.0001

	Straight			Left			Right		
Variable-ROMs	Est	SE	P	Est	SE	P	Est	SE	P
FE withers - T15 - tuber sacrale	-0.73	0.048	<0.0001	-0.85	0.055	<0.0001	-0.56	0.049	<0.0001
FE withers - T12 - T15	-0.43	0.052	<0.0001	-0.56	0.060	<0.0001	-0.16	0.053	0.003
FE T12 - T15 - T18	-0.37	0.034	<0.0001	-0.09	0.039	0.02	0.02	0.035	0.58
FE T15 - T18 - L3	-0.19	0.034	<0.0001	-0.05	0.040	0.20	-0.03	0.035	0.35
FE T18 - L3 - L5	-0.12	0.031	0.0001	0.27	0.036	<0.0001	0.11	0.032	0.001
FE L3 - L5- tuber sacrale	-0.06	0.035	0.06	-0.02	0.040	0.57	-0.25	0.035	<0.0001
FE L5 - tuber sacrale - S5	-0.09	0.043	0.04	0.34	0.050	<0.0001	-0.31	0.044	<0.0001
LB withers - T15 - tuber sacrale	0.89	0.072	<0.0001	0.03	0.083	0.75	0.47	0.074	<0.0001
LB withers - T12 - T15	1.31	0.085	<0.0001	0.89	0.099	<0.0001	0.58	0.088	<0.0001
LB T12 - T15 - T18	0.39	0.071	<0.0001	0.35	0.083	<0.0001	0.35	0.073	<0.0001
LB T15 - T18 - L3	-0.23	0.053	<0.0001	-0.16	0.061	0.01	0.09	0.054	0.11
LB T18 - L3 - L5	0.05	0.052	0.31	0.21	0.061	0.001	0.47	0.054	<0.0001
LB L3 - L5- tuber sacrale	0.55	0.047	<0.0001	1.08	0.055	<0.0001	0.80	0.049	<0.0001
LB L5 - tuber sacrale - S5	0.28	0.049	<0.0001	0.39	0.056	<0.0001	0.16	0.050	0.002
Pelvis roll	0.34	0.124	0.007	0.85	0.143	<0.0001	0.87	0.127	<0.0001
Pelvis pitch	-0.49	0.060	<0.0001	-0.53	0.070	<0.0001	-0.47	0.062	<0.0001
Pelvis yaw	-0.35	0.050	<0.0001	-0.16	0.058	0.005	-0.35	0.052	<0.0001

Supplementary Table S2.

Mixed models where the dependent variable is stride mean lateral bending, stride range of motion (ROM) lateral bending, stride mean flexion-extension and stride ROM flexion extension (top to bottom), and independent variables (fixed effects) as listed in the leftmost column. Models are based on data from 12 horses measured on the straight line (n=1338 observations (strides) and left (n=3260 observations) and right circles (n= 3457 observations). Models were made separately for each path.

Stride mean lateral bending		Left			Straight			Right	
Independent variables	Est	SE	P	Est	SE	P	Est	SE	P
Intercept	-3.56	0.863	0.001	-3.55	0.827	0.0004	-2.29	0.875	0.02
Pelvis roll mean	0.02	0.003	<0.0001	0.04	0.007	<0.0001	0.08	0.003	<0.0001
Pelvis pitch mean	-0.01	0.007	0.46	0.04	0.011	0.0002	0.03	0.007	0.0002
Pelvis yaw mean	1.37	0.011	<0.0001	1.11	0.021	<0.0001	1.30	0.012	<0.0001
Head swivel mean	0.05	0.001	<0.0001	0.06	0.002	<0.0001	0.05	0.001	<0.0001
Body tracking mean	0.02	0.003	<0.0001	-0.02	0.007	0.007	-0.02	0.003	<0.0001
Speed	0.19	0.038	<0.0001	-0.13	0.040	0.001	-0.61	0.039	<0.0001
Stride ROM lateral bending		Left			Straight			Right	
Independent variables	Est	SE	P	Est	SE	P	Est	SE	P
Intercept	3.47	0.413	<0.0001	4.38	0.558	0.003	2.40	0.451	<0.0001
Pelvis roll ROM	-0.14	0.008	<0.0001	-0.10	0.014	<0.0001	-0.08	0.007	<0.0001
Pelvis pitch ROM	0.00	0.019	0.85	0.01	0.026	<0.0001	0.05	0.016	0.002
Pelvis yaw ROM	0.90	0.022	<0.0001	0.27	0.026	<0.0001	0.68	0.019	<0.0001
Head swivel mean	-0.04	0.002	<0.0001	0.01	0.005	0.82	-0.02	0.003	<0.0001
Body tracking mean	-0.01	0.009	0.43	-0.06	0.020	<0.0001	-0.05	0.008	<0.0001
Speed	0.51	0.076	<0.0001	0.80	0.092	<0.0001	0.75	0.078	<0.0001
Stride mean flexion extension		Left			Straight			Right	
Independent variables	Est	SE	P	Est	SE	P	Est	SE	P
Intercept	-12.70	0.858	<0.0001	-22.65	1.112	<0.0001	-16.11	0.910	<0.0001
Pelvis roll mean	0.04	0.010	<0.0001	0.00	0.014	0.98	-0.04	0.009	<0.0001
Pelvis pitch mean	-0.08	0.020	<0.0001	0.19	0.023	<0.0001	-0.02	0.019	0.39
Pelvis yaw mean	0.00	0.024	0.97	0.04	0.029	0.15	0.18	0.022	<0.0001
Head swivel mean	-0.03	0.003	<0.0001	-0.03	0.005	<0.0001	-0.04	0.003	<0.0001
Body tracking mean	-0.08	0.010	<0.0001	0.01	0.018	0.45	-0.11	0.009	<0.0001
Speed	-0.24	0.115	0.04	-0.58	0.090	<0.0001	-0.20	0.113	0.08
Stride ROM flexion extension		Left			Straight			Right	
Independent variables	Est	SE	P	Est	SE	P	Est	SE	P
Intercept	4.69	0.313	<0.0001	5.02	0.332	<0.0001	5.77	0.313	<0.0001
Pelvis roll ROM	0.03	0.007	<0.0001	0.02	0.009	0.10	0.05	0.005	<0.0001
Pelvis pitch ROM	0.29	0.015	<0.0001	0.20	0.018	<0.0001	0.19	0.012	<0.0001
Pelvis yaw ROM	0.13	0.018	<0.0001	0.00	0.018	0.86	0.12	0.015	<0.0001
Head swivel mean	0.01	0.002	<0.0001	0.03	0.004	<0.0001	0.00	0.002	0.57
Body tracking mean	-0.01	0.007	0.06	-0.03	0.013	0.03	0.00	0.006	0.92
Speed	-0.71	0.059	<0.0001	-0.43	0.063	<0.0001	-0.86	0.058	<0.0001

Supplementary Figure S1.

Illustration of marker placement. Markers were placed on the dorsal spinous processes of T12, T15, T18, L3, L5 and S5. Strips with three markers were placed on the forehead (the lowest marker was used in the analysis), and on the withers (the marker on the highest point was used). A T-shaped strip was fitted to the tubera sacrale and the craniodorsal aspects of both tubera coxae.

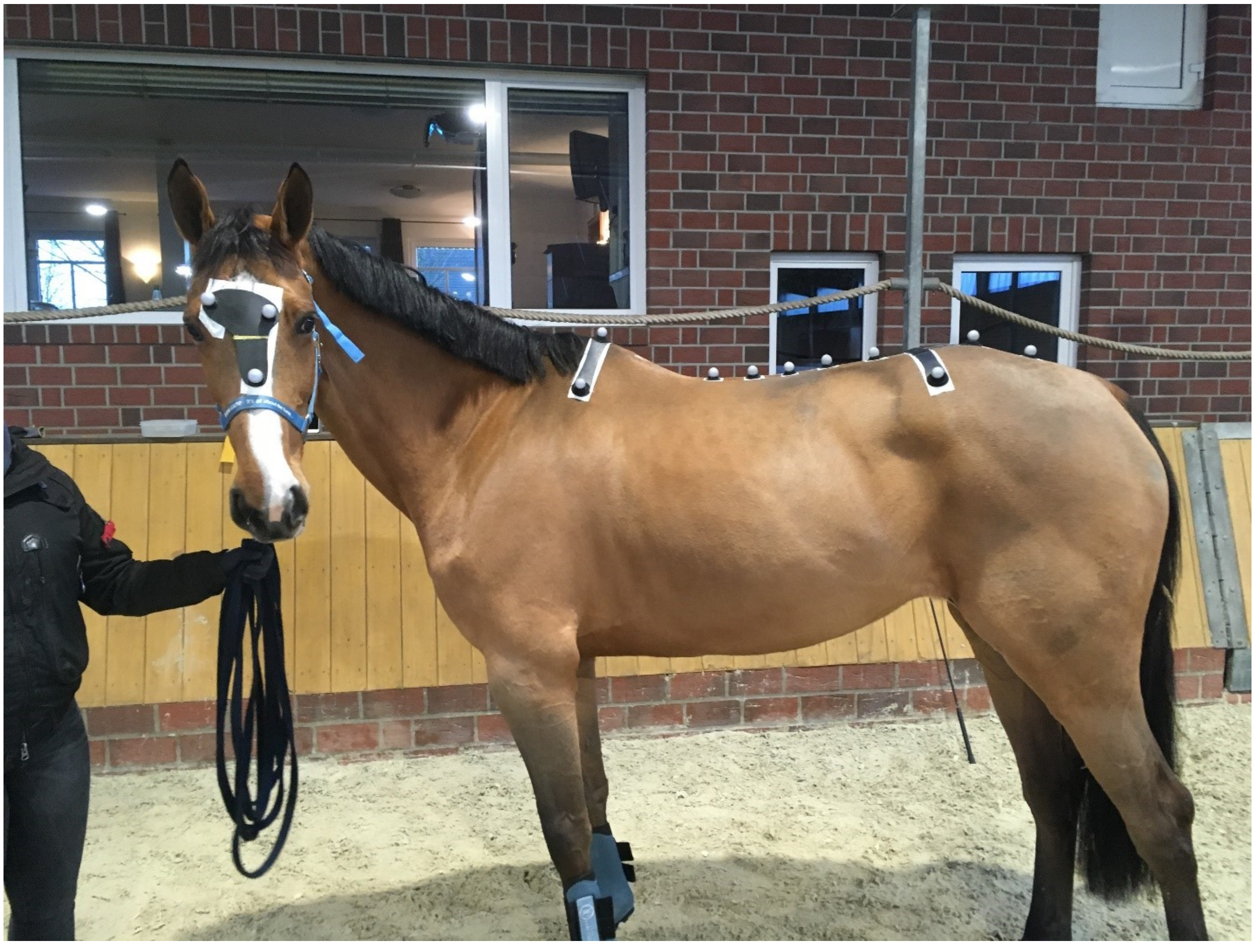

Supplementary Figure S2.

Illustration of the back angle calculations, the flexion-extension angle for the back segment T12 (withers - T12 -T15) is shown as an example. Flexion-extension angles were calculated in the vertical plane (seen from the horse's side as shown) and lateral bending angles were calculated principally the same but in the horizontal plane ('seen from above'). Back segment angles were determined between each set of three markers (withers - T12 -T15; T12-T15-T18; etc., markers used are highlighted in yellow). Whole back angles were determined between the markers at the highest point of the withers, T15 and tubera sacrale (the next most caudal of the highlighted markers). Flexion-extension angles were defined as zero if the set of three markers were level, positive for flexion of the back and negative for extension (extension is illustrated). Lateral bending angles were defined following the same principle: zero when markers were aligned in the sagittal plane, positive for bending of the back to the right and negative for bending to the left.

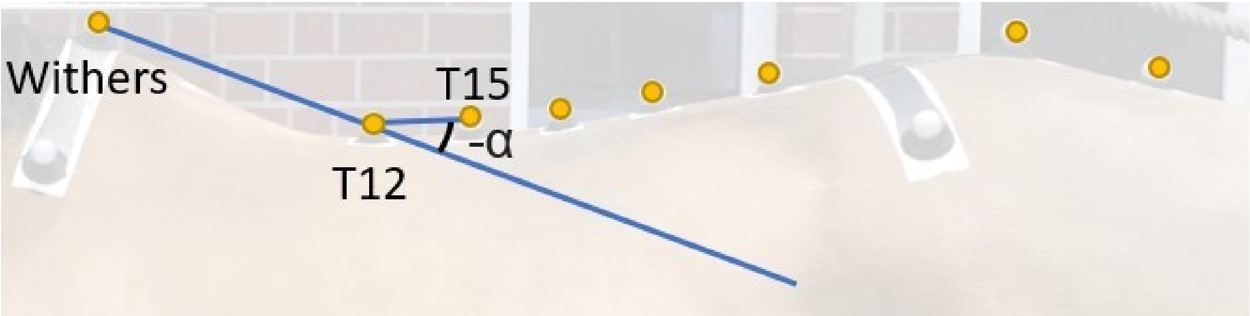

Supplementary Figure S3.

Pelvic rotations plotted versus whole back variables. These pelvic rotation variables were selected for plotting because they showed the largest estimate in multivariable models with the respective whole back variable as the dependent variable, see Supplementary Table S2. Each plot contain data from 12 horses (each horse has a different symbol) measured on three paths (left, right circles and straight line, path not indicated), during two different days on five occasions each day (n= 355 measurements in total, each symbol/data point represents one measurement).

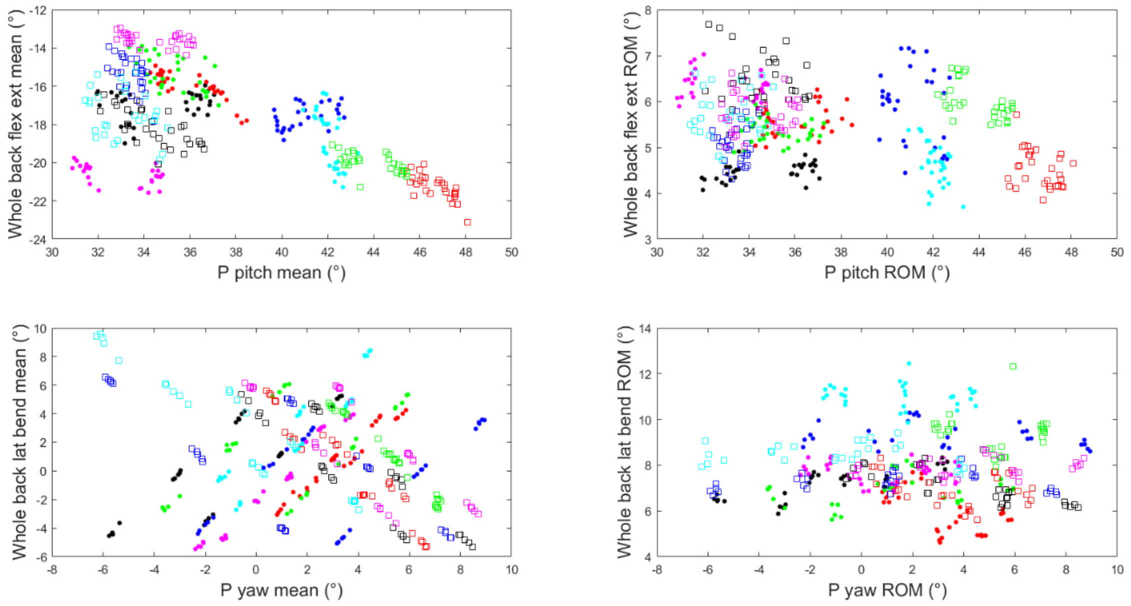

Supplement: Supplementary file 1 — Supplementary Information 1. [file 41598_2021_92272_MOESM1_ESM.pdf]
